# Intermittent Hypoxia Mimicking Sleep Apnea Induces Systemic and Tissue Specific Epigenetic Changes and p16-Mediated Cellular Senescence Underlying Vascular Dysfunction

**DOI:** 10.21203/rs.3.rs-9371471/v1

**Published:** 2026-04-15

**Authors:** Rene Cortese, Kylie Cataldo, Mohammad Badran, Milda Milciute, Juozas Gordevicius, Zhuanghong Qiao, Jonathan Eusey, Abdelnaby Khalyfa, David Gozal

**Affiliations:** University of Kansas Medical Center; University of Missouri; University of Missouri; Vugene; Vugene; University of Missouri; University of Kansas Medical Center; Marshall University; Marshall University

**Keywords:** Obstructive Sleep Apnea, Intermittent Hypoxia, Cellular Senescence, Epigenetic Age Acceleration, Vascular Dysfunction

## Abstract

**Background:**

Obstructive Sleep Apnea (OSA) is a pervasive cardiovascular risk factor linked to accelerated aging and systemic inflammation. Intermittent hypoxia (IH)—a hallmark of OSA—induces cardiovascular decline, yet the underlying tissue-specific and systemic epigenetic mechanisms and the role of cellular senescence in the pathophysiology of OSA and associated cardiovascular disease (CVD) remain poorly understood.

**Methods:**

C57BL/6J male mice were exposed to IH or room air (RA) for durations ranging from 7 to 210 days. Genome-wide DNA methylation profiling was conducted on left cardiac ventricle and peripheral blood mononuclear cells (PBMCs) samples. Differentially methylated positions (DMP; q<0.05) were identified between IH and RA groups and the impact of IH duration was studied using regression models. Epigenetic age acceleration (EAA) was calculated using a multi-tissue epigenetic clock. Furthermore, p16-reporter and targeted ablation mouse models (p16-Cre^ERT2^-DTR-tdTomato) were utilized to assess the role of p16^Ink4a^-mediated senescence in IH-induced vascular dysfunction.

**Results:**

Chronic IH exposures significantly increased systolic and diastolic blood pressure and altered endothelial function. Epigenetic analysis identified 5,747 and 1,307 DMLs in the left cardiac ventricle and PBMCs, respectively, with minimal overlap between tissues (n=163, p=8.03 × 10^−8^; Fisher’s Exact Test), indicating a highly tissue-specific epigenetic response. Both tissues exhibited an early peak in EAA at 7 days of exposure, differing in the trajectory in longer exposures. Pathway analysis linked these epigenetic changes to cardiac dysfunction and cellular senescence, specifically highlighting *Cdkn2a* gene, which encodes the p16 protein, a marker of cellular senescence. Immunofluorescence confirmed increased p16 expression in aortic endothelial cells following IH. Remarkably, systemic ablation of p16-expressing cells (i.e., p16^high^ cells), reversed IH-induced hypertension and restored coronary flow reserve to control levels.

**Conclusions:**

IH induces duration-dependent, tissue-specific epigenetic dysregulation and accelerated biological aging. Our findings provide initial evidence that p16^Ink4a^-mediated cellular senescence is a primary driver of OSA-induced cardiovascular morbidity and that targeting the senescent endothelium can revert vascular dysfunction, thereby establishing a novel mechanistic framework for cellular senescence as a therapeutic target in OSA.

## Introduction

Obstructive Sleep Apnea (OSA) is widely recognized as an independent and prominent cardiovascular risk factor, associated with incident obesity, atherosclerosis, hypertension (HTN), endothelial dysfunction, arrhythmias, stroke, coronary artery disease (CAD) and heart failure. Severe OSA (i.e., elevated Apnea-Hypopnea Index, AHI as a reporter of respiratory disturbance, evidence of sleep fragmentation and high hypoxic burden) is associated with CAD, vascular dysfunction, arrhythmias, and heart failure^1–3^. Furthermore, OSA is also associated with metabolic disorders, which are themselves tightly associated with cardiovascular pathologies^4^. Both epidemiologic and intervention-based studies have provided conclusive evidence indicating a causative link between OSA and cardiovascular morbidity, as recently reviewed in^4^. Most OSA morbidities reflect activation and propagation of oxidative stress and systemic inflammatory pathways in a variety of conditions^5–10^, and essentially mimic accelerated aging and cellular senescence^11,12^. However, the molecular, epigenetic and cellular mechanisms linking OSA and cardiovascular disease (CVD) are still poorly understood and have not been systematically explored.

Pre-clinical studies using animal models have consistently demonstrated a major role for intermittent hypoxia (IH) in cardiovascular events, inducing hypertension, pro-atherogenic aortic remodeling and early cardiac remodeling in young rodents^13–15^. Our group has recently reported that mice exposed to 22 weeks of IH (i.e., modeling lifelong OSA) exhibited higher mortality and exacerbated cardiovascular decline when compared to the findings normally associated with aging^16^. Furthermore, the prevalence of OSA increases with aging^17^. It has been suggested that OSA might anticipate/aggravate aging by inducing cellular and molecular impairments that characterize the aging process^11^. During aging, there is a continuous accumulation of epigenetic changes, which might give rise to multiple age-related pathologies, and arise systemically or restricted to a specific tissue/cell type. We have previously demonstrated that OSA patients present systemic epigenetic age acceleration (i.e., measured in blood samples) compared with no-OSA controls matched by chronological age, sex, and BMI^18^. Remarkably, in a follow-up visit a year later, OSA patients showed a de-acceleration of the epigenetic age (possibly and likely due to treatment), whereas the epigenetic age acceleration trends were maintained in non-OSA individuals^18^. This evidence supports the idea of an OSA-associated age acceleration, although the cellular and molecular mechanisms are still to be elucidated.

Importantly, oxidative stress and inflammation have a central role in cardiovascular aging (reviewed in^19^). Excess reactive oxygen species (ROS) and superoxide generated by oxidative stress and low-grade inflammation accompanying aging recapitulate age-related cardiovascular dysfunction^19^. OSA leads to altered oxidative stress and inflammatory pathways in several organs including the cardiovascular system^20^, suggesting that OSA induces aging-related cellular and molecular dysfunctions in CVD phenotypes. Cellular senescence, defined as a cessation of cell division of previously proliferation-competent cells, is implicated in aging-related diseases including CVD^21,22^. We previously reported accelerated vascular senescence induced by OSA and showed that exosomes from untreated OSA patients induced significant increases senescence markers, including x-gal and p16(CDKN2A)^22^. Hence, OSA may initiate and exacerbate cellular aging in endothelial cells, either directly or indirectly via exosomes, and such accelerated senescence may be only partially reversible upon long-term adherent CPAP treatment^18^.

In the present study, we hypothesized that exposures to IH, simulating OSA, will induce systemic and tissue-specific epigenetic changes that are associated with accelerated biological aging and increased senescence of vascular cells resulting in cardiovascular dysfunction. By exposing mice to different durations of IH exposures, we aimed to identify key epigenetic changes occurring specifically in the cardiovascular tissue and at the systemic level. Furthermore, we took advantage of p16 reporter mouse models (p16-Cre^ERT2^-tdTomato)^23^ to investigate the phenotypic effects and molecular mechanisms of IH-induced cardiovascular senescence, and their reversibility through selective targeting of senescent cells in the vasculature.

## Materials and Methods

### Animals and IH exposures.

The protocol for animal work was reviewed and approved by the IACUC at the Kansas University Medical Center (Protocol #IPROTO202500000172). For the study of epigenetic changes associated with different lengths of IH exposure, C57BL/6J male mice (7-week old) were acquired from Jackson Laboratories (Bar Harbor, ME). Mice were divided into two groups (n=25 mice/group) and exposed to IH (i.e., 21% FIO_2_ and 6.1% FIO_2_; 20 cycles/h for 12 h/day) or room air (RA, continuous 21% FiO_2_) conditions for 7,14, 30, 120 and 210 days. These exposures simulate the recurrent oxyhemoglobin desaturations commonly observed in patients suffering of moderate to severe OSA^24^. At each time point, n=5 animals/group were sacrificed, and blood and left cardiac ventricle samples were collected. Blood samples were stored at −80 C until use. Tissue samples were flash frozen in liquid nitrogen and stored at −80 °C until use.

p16-Cre^ERT2^-tdTomato and p16-Cre^ERT2^-tdTomato mice used for the *in vivo* assessment and ablation of p16^high^ cells were a gift from Dr. Nakanishi (Institute of Medical Science, University of Tokyo, Japan). In brief, p16-Cre^ERT2^-tdTomato mice were generated by inserting Cre^ERT2^ into the endogenous p16-Ink4a locus and intercrossing these mice with Rosa26-CAG-lsl-tdTomato mice^23^. These reporter mice enable *in vivo* detection of p16^high^ cells upon transgene activation (Figure 5A). p16-Cre^ERT2^-DTR tdTomato mice were generated by intercrossing the Cre^ERT2^-p16-Ink4a mice with Rosa26-SA-lsl-DTR-IRES-tdTomato mice^23^. These double-transgenic mice contain the DT receptor and the exogenous p16 loci, enabling the systemic ablation of p16^high^ cells by DT injection (Figure 5B). p16-Cre^ERT2^-tdTomato and p16-Cre^ERT2-^tdTomato mice were exposed to IH or RA for 6 weeks, as detailed above. Transgene was induced in all animals by daily intraperitoneal (i.p.) injection with tamoxifen (TAM, 80 mg/kg BW) for five days during the first week, as detailed in^23^. A new round of TAM induction (i.e., 80 mg/kg BW i.p. daily for five days) was conducted at week 3. In p16-Cre^ERT2^-DTR-tdTomato systemic ablation of p16^high^ cells was achieved by treatment with Diphtheria Toxin (DT). After preliminary dosage optimization procedures, low dose DT was administered to RA- and IH-exposed mice (RA/DT+ and IH/DT+ groups, n=8 mice/group), whereas control groups received only vehicle (RA/DT− and IH/DT− groups, n=8 mice/group). After RA or IH exposures were completed, mice were sacrificed, and blood and tissue samples were collected.

### Sample preparation and processing for molecular studies:

At each pre-determined time point tissue samples were collected after euthanasia of the animals in each experimental group. Blood was drawn from each mouse by cardiac puncture, the plasma and cellular fractions were separated by centrifugation, aliquoted and stored at −80°C. Left cardiac ventricle were carefully dissected, snap frozen in liquid nitrogen, and stored at −80°C. Genomic DNA was isolated from peripheral blood lymphocytes using the DNeasy Blood & Tissue kit (Qiagen, Valencia, CA). DNA and samples were stored at −80 °C until use.

### Blood pressure measurements, coronary artery blood flow reserve and endothelial stiffness.

Heart rate and arterial blood pressure were measured using a non-invasive approach by the tail cuff method by volume pressure recording (CODA system—Kent Scientific, Torrington, CT, USA) in conscious animals between noon and 1:00 pm. Mice were placed in a cylindrical holder over a warmed blanket. After 30 minutes of habituation, at least 8 recordings were performed, each separated by 5 minutes. For data analysis, we considered the mean of the lowest five values for Systolic (SAP), Diastolic (DAP), and Mean (MAP) Blood Pressure^25^. Atomic Force Microscopy (AFM) was used to evaluate *ex vivo* endothelial stiffness in *en face* vessel preparations^26^ and isolated endothelial cells. Coronary Flow Velocity Reserve (CFVR) was assessed by the method described in^27^ and expressed as expressed as hyperemia/baseline ration in response to increased isoflurane concentration. For isolated cell experiments, coronary arteries were dissociated, and AFM measurements will be performed midway between the cell perimeter and the nucleus to limit recording to changes in cortical stiffness. Force curves were obtained using a nanoindentation protocol using an MFP-3D AFM (Asylum Research, Goleta, CA) mounted on an Olympus IX81 microscope and using Igor Pro software (version 6.37). The measurements were made at room temperature (~25°C). A total of at least 30 curves will be obtained per cell per coronary artery explant site and repeated for at least five cells per site per sample. Elastic moduli were calculated from the force curves using a custom-made Python script that in an unbiased manner identifies and removes curves with excessive noise, and the software selected the first 15 curves from the remaining ones, and they were fitted to the Hertz model of a conical tip, as previously described^28^.

### Microarray-based epigenetic profiling.

For studying Genome-wide DNA methylation profiles, 1 μg of genomic DNA was treated with sodium bisulfite (Zymo Research, Irvine, CA). Converted DNA was analyzed using Infinium Mouse Methylation BeadChip assay (Illumina, San Diego, CA). This array includes over 285,000 CpG sites covering all RefSeq genes, including CpG islands, translation start sites, enhancers, imprinted loci, and other regions^29^. The dataset is available at the NCBI Gene Expression Omnibus (GEO) repository (accession number pending). All data analyses were conducted using the R environment version 4.2.0. Microarray data was processed using the *ENmix* version.1.34.02^30^ and *minfi* v.1.44.0^31^ packages. Quantile normalization of U or M intensities for Infinium I or II probes were performed, respectively. A model-based correction was performed, and a dye-bias correction was conducted using *RELIC*^32^. β-values representing the averaged total intensity value per CG position was calculated as unmethylated intensity (U) + methylated intensity (M) [M / (U + M + 100)]. Probes with a detection p < 1 × 10^−6^ and less than 3 beads were defined as low quality. Samples with low quality methylation measurements > 5% or low intensity bisulfite conversion probes were removed from further analysis. Differentially methylated positions (DMPs) between the experimental groups were determined using the *ENmix* version.1.34.02^30^ package. A regression model was created including exposure type (i.e., RA or IH), exposure time (i.e., 7,14, 30, 120 and 210 days) and Sentrix ID. Empirical Bayes correction was applied to the model fits. For each position and contrast, the magnitude of the DNA methylation difference was expressed as Fold Changes in the logarithmic scale (logFC) and the significance of the difference as FDR-corrected p-Value (q value). Overlaps with biological processes and molecular networks were determined using Ingenuity Pathway Analysis (IPA) software (QIAGEN, Redwood City, CA)^33^. Epigenetic age and acceleration were calculated using multi-tissue full lifespan epigenetic clock^34^.

### Immunofluorescence analysis:

Distal aorta sections were collected after euthanasia and perfused with 1X PBS. Perivascular fat was removed, and sections were embedded in Tissue-Tek OCT compound (Sakura Finetek USA, Torrance, CA) and stored frozen at −80 °C until use. Cryosectioned aortas were permeabilized with 0.2% Triton X-100 in PBS for 5 minutes and incubated in blocking buffer (5% BSA in PBS) for 30 minutes. Samples were then incubated with antibodies against SMA (Cat # ab202296, Abcam) or CD31(Cat # ab56299, Abcam) (1/100 dilution) at room temperature for 2 hours, followed by incubation with anti-rabbit IgG conjugated with Alexa Fluor 633 (Cat # A21070, Thermofisher Scientific) or Alexa Flour Plus 647 (Cat # A48272, Thermofisher Scientific) secondary antibodies for SMA and CD31, respectively, diluted in blocking buffer (1:200) for 1 hour at room temperature. Nuclei were counterstained with DAPI (1:1000) for 5 minutes. Images were acquired using a SP8 – STED spectral confocal microscope (Leica). For each measurement, images from n=8–12 consecutive sections were quantified using the ImageJ software (NIH, Bethesda, MD).

## Results

### Effects of different lengths of IH exposure in vascular function

**Figure 1:**
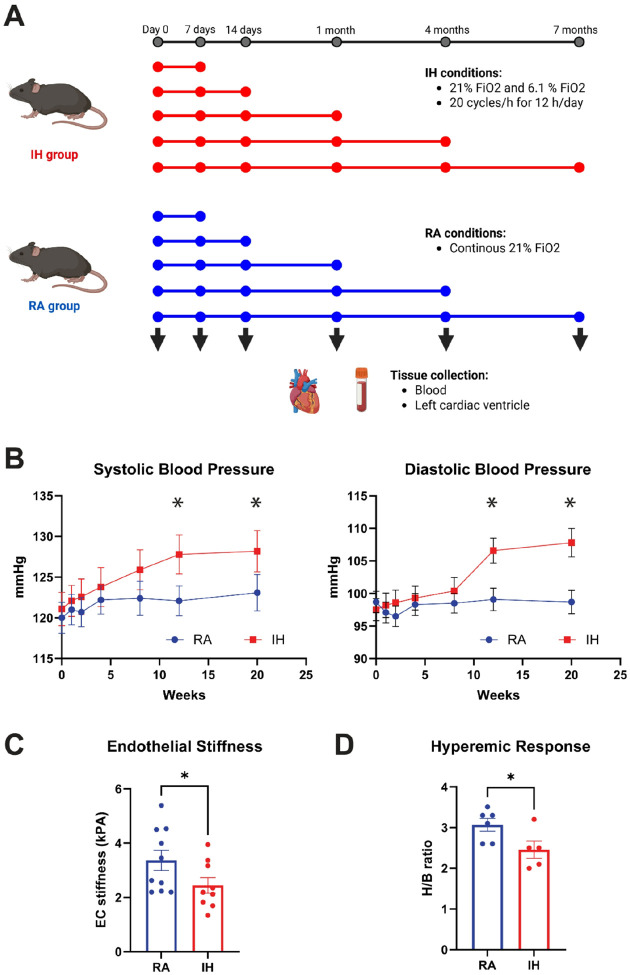
Vascular dysfunction in mice exposed to IH. A) Experimental design: Mice were divided into two groups (n=25 mice/group) and exposed to IH (i.e., 21% FIO_2_ and 6.1% FIO_2_ (nadir SpO_2_ −68–75%); 20 cycles/hour for 12 hour/day) or room air (RA, continuous 21% FiO_2_) conditions for 7,14, 30, 120 and 210 days. At each time point, n=5 animals/group were sacrificed, and samples(i.e., peripheral blood, left cardiac ventricle, and aorta) were collected for further analyses. **B) Increased systolic and diastolic blood pressure in mice exposed to chronic IH.** Systolic and diastolic arterial blood pressure measurements were acquired at weeks 0, 1, 2, 4, 8, 12, and 20 using the tail cuff method. After 20 weeks, systolic and diastolic blood pressure significantly increased in mice exposed to IH (p=0.025 and p=0.001, respectively; Student’s t-test). **C) Reduced aortic endothelial stiffness in mice exposed to IH.** Mice were exposed to IH or RA condition as detailed in A for 4 weeks. A total of at least 30 force curves were obtained per cell per aortic explant site and repeated for at least five cells per site per sample. Aortic stiffness was reduced in mice exposed to IH (p = 0.03). **D) Reduced CFVR in mice exposed to IH.** Mice were exposed to 6 weeks of IH or RA conditions, coronary artery baseline flow velocity was recorded at 1% isoflurane concentration. The hyperemic flow velocity was recorded during maximal vasodilatation induced by 2.5% isoflurane, and after three minutes of isoflurane inhalation. CFVR was calculated as the ratio of hyperemic peak flow velocity to baseline peak flow velocity (H/B). IH exposures for 6 weeks significantly reduced hyperemic responses to 2.5% isoflurane (p<0.009).

Differences in IH exposure duration promote emergence of tissue-specific phenotypic differences with activation of numerous genes and molecular pathways^35,36^. To study the effects of different IH exposure times in the molecular physiology and function of the cardiovascular system, we exposed mice to IH or RA conditions for 7,14, 30, 120 and 210 days. At each timepoint, n=5 animals per group were sacrificed, and cardiac tissue and Peripheral Blood Mononuclear Cells (PBMCs) were collected (Figure 1). Systolic and diastolic arterial blood pressure measurements were acquired at weeks 0, 1, 2, 4, 8, 12, and 20. After 20 weeks, systolic blood pressure significantly increased in mice exposed to IH (p=0.025; Student’s t-test), with minimal changes in the control group (p=0.237, Student’s t-test). Similarly, diastolic blood pressure significantly increased in mice exposed to IH (p=0.001, Student’s t-test, whereas the control group exhibited no differences (p=1, Student’s t-test) (Figure 1B). These results indicate that chronic exposures to IH increase arterial blood pressure, which may eventually contribute to altered vascular stiffness and endothelial dysfunction^37^. Hence, we verified whether IH exposure led to reduced aortic endothelial stiffness in mice. Aortic stiffness was significantly reduced in mice exposed to IH compared to controls (p = 0.03, Student’s t-test) (Figure 1C). Furthermore, IH exposures significantly attenuated coronary artery hyperemic responses to 2.5% isoflurane, reflecting significant reductions in CFVR in IH-exposed mice compared to controls (2.5 ± 0.3 vs 3.2 ± 0.2, for IH and RA groups, respectively; p= 0.009) (Figure 1D), thereby indicating that IH induces coronary artery dysfunction.

### IH exposure alters cardiac and systemic epigenetic profiles affecting pathways related to aging and cellular senescence.

**Figure 2:**
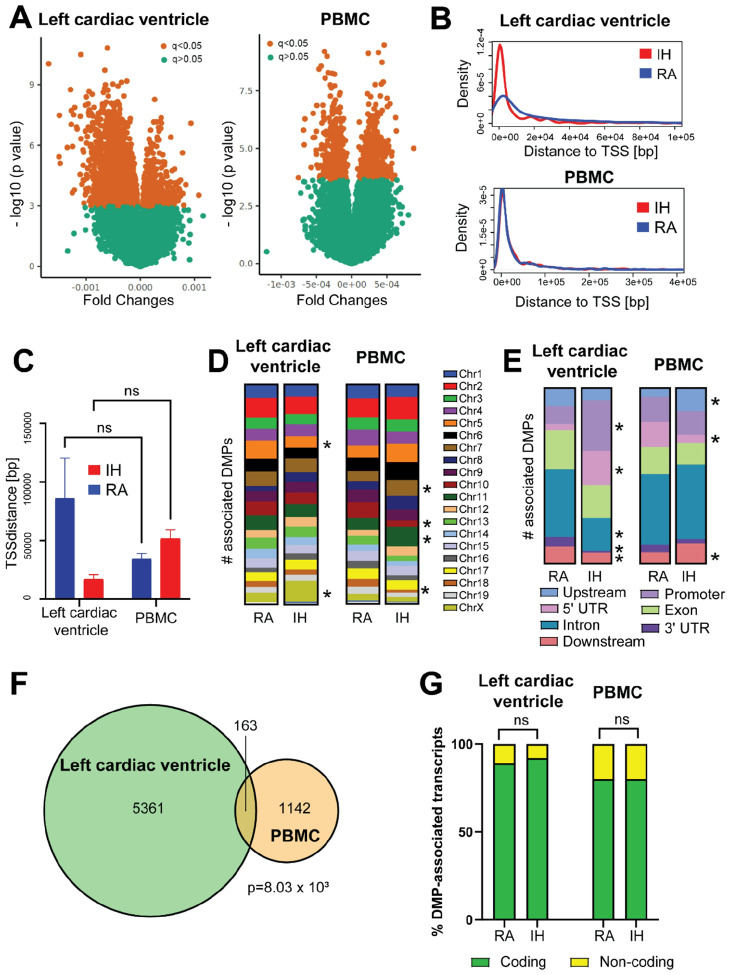
IH exposure leads to systemic and tissue-specific changes epigenetic changes. **A)** A linear regression model was built including type of exposure (i.e., IH or RA) and length of exposure (i.e., 7d, 14d, 30d, 120d and 210d) as covariates. The regression model enabled the identification of tissue-specific DMPs associated with IH exposure in the left cardiac ventricle (n=5,747) compared with systemic differential methylation (n=1,307). Microarray data is presented as volcano plot for left cardiac ventricle and PBMC samples (left and right panel, respectively). The x- axis represents the magnitude of the difference in signal intensity between the IH and RA groups for each probe in the microarray, expressed as fold changes in log2 scale. Probes with increased microarray signals in IH and RA groups had positive and negative values on the x axis, respectively. The y axis represents the significance of the difference in signal intensity between the IH and RA groups for each probe in the microarray, expressed as the −log10-transformed p values. Red dots highlight DMPs with significant differences between the IH and RA groups (q-value <0.05). **B)** Density plots showing the distance of the DMPs to the nearest TSS in left cardiac ventricle and PBMC samples. We did not detect significant differences between the IH and RA either in left cardiac ventricle (upper panel) nor in PBMCs (lower panel). The distance from the DMP to the closest TSS is shown on the x axis. Red and blue lines represent the IH and RA groups, respectively. **C)** ANOVA revealed no interaction between the effects of the tissue type (i.e., left cardiac ventricle and PBMC) and exposure (i.e., RA and IH) on the DMP distance to TSS (p = 0.556). IH and RA groups in each tissue type are represented by red and blue columns, respectively. **D)** DMP mapping in mouse genome by chromosome. Significant DMP overrepresentation (p<0.05) in chromosomes 5 and X, and in chromosomes 7,10, 11, and 18 in left cardiac ventricle and PBMC samples, respectively. Chromosomes with DMP overrepresentation in each tissue are marked with an *. **E)** DMP association with gene regions. DMPs highly methylated in IH compared to the RA group were overrepresented in the promoter, 5’UTR, intronic, 3’UTR and downstream regions in left cardiac ventricle, and in the upstream and 5’UTR regions in PBMC samples, respectively. Gene regions with DMP overrepresentation in each tissue are marked with an * **F)** Overlap between DMP-associated transcript in left cardiac ventricle and PBMC. Only a non-significant fraction of DMP-associated genes overlapped between the tissue (n=163, p=8.03 × 10–8; Fisher’s Exact Test). G) Association with coding (green column portions) and non-coding (yellow column portions) did not significantly differ between DMPs with higher DNA methylation in the IH or RA groups neither in left cardiac ventricle nor in PBMC samples. ns=non-significant (p>0.05; Fisher’s exact test).

To further investigate the tissue-specific and systemic epigenetic mechanisms by which IH alters cardiovascular function, we conducted large-scale DNA methylation profiling in cardiac tissue (i.e., left ventricle) and PBMC of mice exposed to IH for 7, 14, 30, 120 and 210 days (Figure 2). We built linear regression models for each tissue including type of exposure (i.e., IH or RA) and length of exposure (i.e., 7, 14, 30, 120 and 210 days) as covariates. The regression model enabled the identification of tissue specific Differentially Methylated Positions (DMPs, q<0.05) associated with IH exposure in the left cardiac ventricle (n=5,747, with n=465 and n=5,282 highly methylated in IH and RA groups, respectively; Supplementary Table S1) and systemic differential methylation in PBMCs (n=1,307, with n=926 and n=381 highly methylated in IH and RA groups, respectively; Supplementary Table S2) (Figure 2A). No significant differences emerged between the IH and RA groups in the distance of the DMPs to the transcription start site of the nearest gene neither in left cardiac ventricle (mean distance: IH=17,402.78 ± 3,418.15 bp; RA=86,174.33 ± 34,179.29 bp; p=0.556; t=0.584; 95% CI, −16,941 to 299,485; paired t test) nor in PBMCs (mean distance: IH=52.068.42 ± 7,237.84 bp; RA=34,617.24 ± 4,496.98 bp; p=0.109; t=1.602; 95% CI, −39,827 to 3,294; paired t test) (Figure 2B). Moreover, we did not detect significant interactions between the effects of the tissue type (i.e., left cardiac ventricle and PBMC) and exposures (i.e., RA and IH) on the DMP distance to TSS (F(1,4461) = 0.3462, p = 0.556) (Figure 2C). DMPs were mapped across all genome and gene regions. We observed an overrepresentation of DMPs highly methylated in IH compared to the RA group in chromosomes 5 and X in cardiac left ventricle samples, and in chromosomes 7,10, 11, and 18 in PBMC samples (p<0.05, Fisher’s exact test; Figure 2D, Supplementary Table S3). Likewise, DMPs highly methylated in IH compared to the RA group were overrepresented in the promoter, 5’UTR, intronic, 3’UTR and downstream regions in left cardiac ventricle, and in the upstream, 5’UTR and downstream regions in PBMC samples (Figure 2E, Supplementary Table S4). DMPs in the were associated to n=3,831 and n=977 annotated transcripts (Genome Reference Consortium, build 39 / mouse genome version 39 [GRCm39/mm39] assembly) in left cardiac ventricle and PBMCs, respectively. Only a non-significant fraction of DMP-associated genes overlapped between left cardiac ventricle and PBMC (n=163, p=8.03 × 10^−8^; Fisher’s Exact Test), denoting tissue-specific epigenetic dysregulation (Figure 2F). In left cardiac ventricle, n=3,419 and n=412 DMP-associated transcripts corresponded to protein coding and non-coding transcripts, respectively. Likewise, in PBMCs n=782 and n=195 DMP-associated transcripts corresponded to protein coding and non-coding transcripts, respectively. No significant differences were detected in the proportion of DMPs associated to coding or non-coding transcripts between IH and RA groups neither in left cardiac ventricle (p=0.063) nor in PBMC samples (p=0.8664) (Figure 2G).

**Figure 3:**
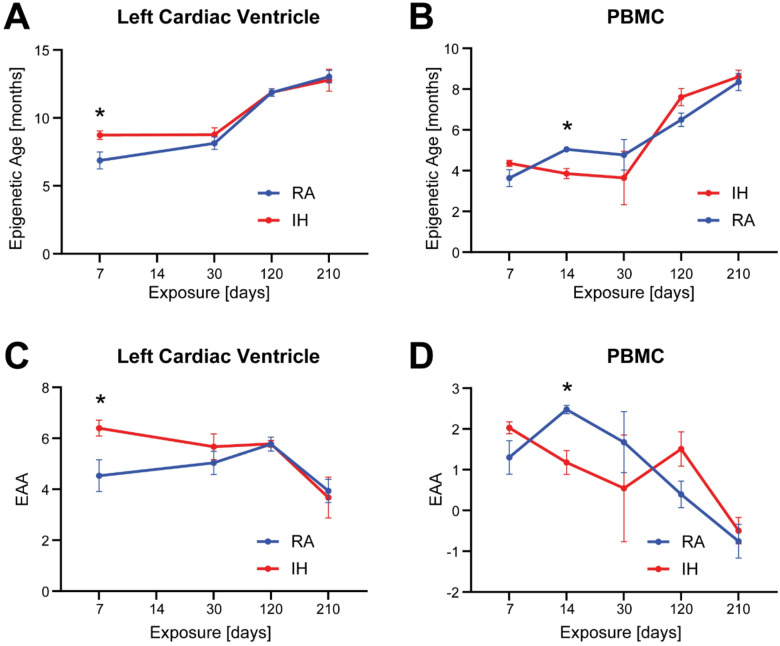
IH-induced systemic and tissue-specific aging. Systemic and tissue-specific epigenetic age and epigenetic age acceleration varied between left cardiac ventricle and PBMC, representing systemic epigenetic age, across tissues according to the length of the exposure. Epigenetic age **(panels A-B)** and Epigenetic Age Acceleration (EAA) **(panels C-D)** were calculated using epigenetic clocks^34^ in samples collected from mice exposed to 7, 14, 30, 120 and 210 days in IH (red lines) or RA (blue lines) conditions, respectively.

Next, to understand how IH exposure impacts epigenetic age in cardiac tissue as well as systemically (i.e., assessed in PBMCs), we calculated epigenetic age and epigenetic age acceleration in samples from mice exposed to IH for 7, 14, 30, 120 and 210 days and comparing with their corresponding RA controls. The trajectory of the epigenetic age was different in left cardiac ventricle and PBMC samples (Figure 3A–B). In left cardiac ventricle – representing tissue specific epigenetic age - IH induced significantly higher epigenetic age in IH than RA samples at 7 days (mean ages 6.864 ± 0.624 and 8.732 ± 0.308 months for RA and IH groups, respectively, p=0.027, unpaired t-test), and then followed virtually the same trajectory with no significant differences between the groups (i.e., p=0.379, p=0.972, and p=0.787 for 30, 120 and 210 exposure days, respectively, unpaired t-test; Figure 3A). In PBMCs -representing systemic epigenetic age-, we also observed an increase of epigenetic age, yet not statistically significant, at day 7 (mean ages 2.297 ± 0.410 and 3.870 ± 0.145 months for RA and IH groups, respectively, p=0.132, unpaired t-test) in IH compared to RA samples. Remarkably, as epigenetic age steadily increased in RA samples, it significantly decreased in IH samples at 14 days (mean ages 5.043 ± 0.102 and 3.854 ± 0.251 months for RA and IH groups, respectively, p=0.002) and maintained at 30 days (mean ages 4.774 ± 0.748 and 2.332 ± 1.310 months for RA and IH groups, respectively, p=0.531), rising up again at 120 days (mean ages 6.169 ± 0.324 and 7.131 ± 0.422 months for RA and IH groups, respectively, p=0.159), and reaching the same epigenetic age by day 210 (mean ages 8.346 ± 0.415 and 8.604 ± 0.323 months for RA and IH groups, respectively, p=0.653; Figure 3B). Likewise, epigenetic age acceleration also followed a distinct trajectory on left cardiac ventricle and PBMC samples (Figure 3C–D). In left cardiac ventricle, EAA in IH samples were significantly higher at 7 days (p=0.027, unpaired t-test), and then decelerated to the same level than in RA samples with no significant differences between the groups (i.e., p=0.379, p=0.972, and p=0.787 for 30, 120 and 210 exposure days, respectively, unpaired t-test; Figure 3C). In PBMC however (Figure 3D), after 7 days of exposure EAA was non-significantly higher in IH compared to RA samples (p=0.133, unpaired t-test). While IH samples showed a sustained deceleration after 14 days and 30 days, EAA peaked in RA samples after 14 days followed by a smooth decline (p=0.0023 and p=0.531 at 14 and 30 days, respectively). EAA peaked after 120 days (p=0.159) and then declined reaching the same level as RA samples after 210 days (p=0.653)

**Figure 4:**
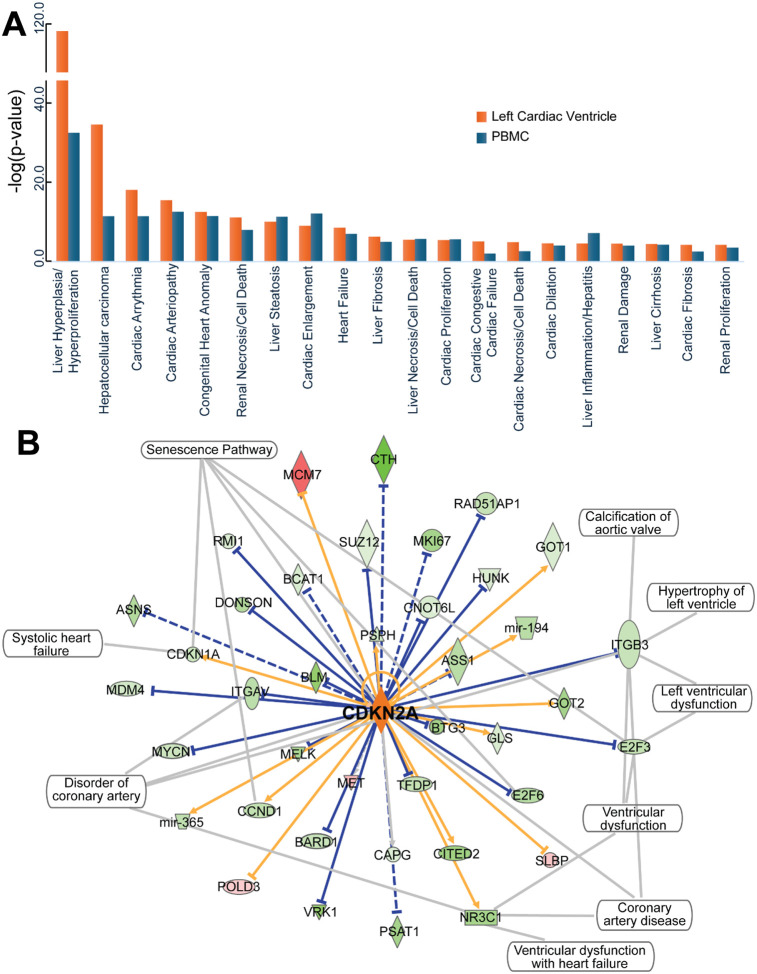
IH induces epigenetic dysregulation of pathways and molecular networks associated with cardiovascular disease and cellular senescence. Biologically relevant gene interaction networks were identified by statistically significant overrepresentation in DMP- associated genes. **A)** Pathophysiological processes associated with genes showing differential DNA methylation between IH and RA groups in left cardiac ventricle (orange bars) and PBMC (blue bars) samples. Bars represent the results of the overrepresentation test [−log10(p-value); hypergeometric test). **B)** Molecular network corresponding to the CDKN2A gene (encoding the p16-protein, a marker of cellular senescence) showing the association with cardiovascular disease. Genes associated with DMPs with higher DNA methylation in IH- and RA-exposed left cardiac ventricle samples are shown in red and green, respectively. Blue and orange lines represent activated and inhibited molecular interactions, respectively. Solid and dashed lines depict experimentally proven and computationally inferred molecular interactions, respectively.

The lists of overrepresented physiological functions related to DMP-associated genes in cardiac left ventricle and PBMCs are provided in Supplementary Table S5. IH-induced epigenetic dysregulation was associated with CVD pathophysiological mechanisms at both the systemic and tissue-specific levels (Figure 4A). For example, ‘Cardiac Arteriopathy” was significantly overrepresented in IH-induced DMPs detected in left cardiac ventricle (p=3.19×10^−16^) and PBMC (p=2.89 ×10^−15^) samples. Further analysis of functional networking between genes associated with IH-induced DMPs identified networks related to cardiac dysfunction, but also to aging and cellular senescence (Supplementary Table S5). Noteworthy, the *Cdkn2a* gene (i.e., encoding the p16 protein, a major regulator of cellular senescence^38^ displayed significantly higher methylation in the IH-exposed left cardiac ventricle compared with the RA group. *Cdkn2a* interacted with other DMP-associated genes related to cardiovascular disease (e.g., *Itgav, Nr3c1,* etc.) and cellular senescence pathways (*Cdkn1a, Ccnd1,* etc.) (Figure 4B) suggesting a major role for the epigenetic dysregulation of this gene and cellular senescence in the pathophysiology of IH-induced cardiovascular dysfunction.

### IH induces p16 expression in endothelial vascular cells leading to vascular dysfunction.

**Figure 5:**
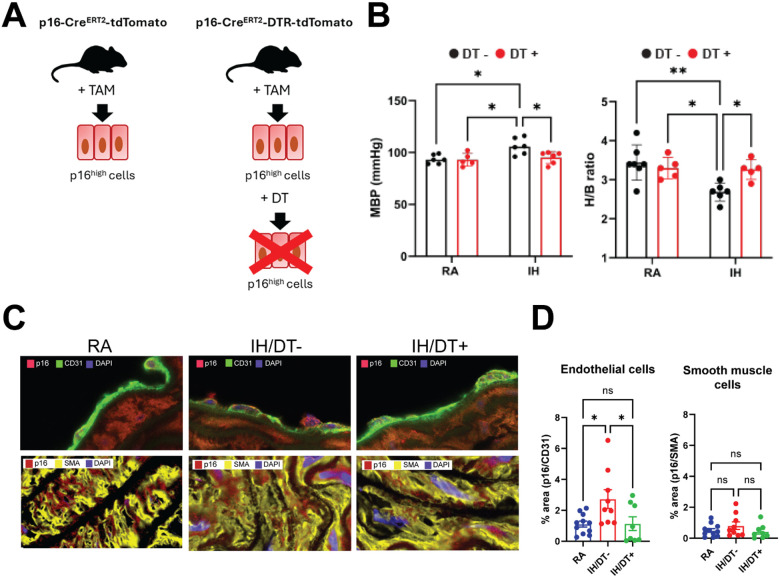
*in vivo* assessment of p16 expression in aorta using p16-Cre^ERT2^-DTR-tdTomato mice. **A)** Transgenic p16-Cre^ERT2^-tdTomato mouse model contains a reporter protein attached to the p16 locus enabling in vivo detection of the p16^high^ cells, upon transgene activation with Tamoxifen (TAM). Transgenic p16-Cre^ERT2^-DTR-tdTomato mouse model also contains a DTR cassette enabling systemic ablation of p16^high^ cells, upon Diphteria Toxin (DT) injection. B) Systemic p16 ablation reverts IH-induced vascular dysfunction. Low dose DT was administered to RA- and IH-exposed mice (RA/DT+ and IH/DT+ groups, n=8 mice/group), whereas control groups received only vehicle (RA/DT− and IH/DT− groups, n=8 mice/group) Ablation of p16^high^ cells significantly reduced mean blood pressure (MBP, left panel) and increased coronary flow reserve - H/B ratio (right panel) (p<0.05; one-way ANOVA; **: p<0.01, *: p<0.05; Tukey test). **C)** IH exposure leads to an increase of p16^high^ cells (red) compared with RA controls. DT treatment leads to ablation of p16^high^ cells. Endothelial and muscle cells are identified with antibodies against CD31 (green, upper panel) and SMA (yellow, lower panel), respectively. Cell nuclei are DAPI stained (blue). 63X magnification. **D)** IH-exposure led to a significant increase of p16 expression only in endothelial cells (p=0.029 and p=0.282, for endothelial and smooth muscle cells, respectively. Two-tailed t-test). p16^high^ cells ablation created a significant difference only in endothelial cells (p=0.0171 and 0.232, for endothelial and smooth muscle cells, respectively. One-way ANOVA). Pairwise differences were determined by post hoc Tukey’s test. *: p<0.05, ns: non-significant.

To further characterize the effects of IH-induced p16-mediated cellular senescence in vascular dysfunction in the distal aorta, we utilized p16 reporter mouse models studying *in vivo* IH-induced p16 expression in aortas (i.e., p16-Cre^ERT2^-tdTomato), as well as systemic ablation of p16^high^ cells upon treatment with Diphtheria Toxin (DT) (p16-Cre^ERT2^-DTR-tdTomato)^23^ (Figure 5A). Mice were exposed to IH or RA for 6 weeks and the transgene was induced by Tamoxifen injection (TAM), as detailed in the materials and methods section. Low dose DT was administered to RA- and IH-exposed mice (RA/DT+ and IH/DT+ groups, n=8 mice/group), whereas control groups received only vehicle (RA/DT− and IH/DT− groups). Ablation of p16^high^ cells significantly reduced mean blood pressure (MBP, Figure 5B) and increased coronary flow reserve - H/B ratio (Figure 5B) (p<0.05; one-way ANOVA with post hoc Tukey test). We confirmed the ablation of p16^high^ cells in aorta tissue of mice exposed to IH and treated with DT by immunofluorescence. Figure 5C illustrates the *in vivo* detection of p16^high^ cells in the aortas of animals in the RA and IH groups. IH-exposure led to an increase of p16 expression in aorta endothelial and smooth muscle cells, with IH/RA differences being statistically significant only in endothelial cells (p=0.029 and p=0.282, for endothelial and smooth muscle cells, respectively; Two-tailed t-test, Figure 5D). Our results confirm a major role of p16 expression in IH-induced vascular dysfunction, warranting further research on the molecular and cellular mechanisms involved, as well as the characterization of the IH-induced epigenetic dysregulation of senescence pathways in vascular cells.

## Discussion

OSA is an extremely prevalent disease that affects virtually all organs and systems. The adverse impact of OSA on cardiovascular function has been repeatedly demonstrated in clinical and epidemiological studies, although disease heterogeneity and conflicting results in clinical trials highlight the need for precise mechanistic studies leading to precision medicine approaches^39–44^. The present study highlights a major role for OSA-induced epigenetic dysregulation and vascular senescence in the pathogenesis of the CVD phenotype. By comparing several exposure durations, we found that the duration of exposure to IH induces systemic and tissue-specific variation in DNA methylation profiles and epigenetic age acceleration, which are significantly associated with increasing blood pressure and reduced endothelial cell stiffness. Analyses of the associated pathways and molecular networks revealed the adverse effects and underlying contributions of senescence in OSA-induced cardiovascular dysfunction, and its role in potential OSA-induced CVD reversibility. By using transgenic mice targeting p16, we assessed the impact of one of the main hallmarks of OSA (i.e., IH) on increasing p16 expression, a key driver of cellular senescence, in endothelial cells and also contributing to blood pressure increases and reduced coronary flow reserve. For the first time, we provided critical evidence showing that systemic ablation of p16^high^ cells reverts IH-induced vascular dysfunction.

### Duration of exposure to IH induces tissue-specific variation in DNA methylation profiles and epigenetic age acceleration associated with cardiovascular dysfunction.

As anticipated from previous studies in our laboratory and by others^16,20^, increasing exposure durations result in augmented blood pressure in IH exposed mice compared with age-matched controls. Noteworthy, differences in both systolic and diastolic pressure steadily increase at short durations and became significant after 10 weeks of exposure while stabilizing thereafter (Figure 1B). Epigenetic regulation tested herein as DNA methylation provides a plausible mechanism underlying this plastic response to the IH stimulus, in which early adaptive responses shift towards a more stable pathological status in the affected tissues^45–47^. In agreement with this hypothesis, we show that IH-induced changes in DNA methylation profiles are tissue-specific, with only a small fraction of DMP-associated genes being actually shared between PBMC and left cardiac ventricle profiles (Figure 3F).

Likewise, we also detected differences between systemic (i.e., PBMC), and tissue-specific (i.e., left cardiac ventricle) epigenetic aging induced by IH exposures. In both instances, we observed an early peak of significantly increased DNAmAge in IH compared to RA, followed by a deceleration in IH samples (Figure 3) reaching the same level as RA. However, while DNAmAge for IH follows the same trajectory as RA samples in left cardiac ventricle, a late DNAmAge peak difference in the systemic age acceleration emerged in IH samples at 120 days of exposure. Differences between tissue-specific and systemic epigenetic aging can be due to several factors. First, as peripheral blood lymphocytes undergo constant division, variations in DNA methylation patterns accumulate with each cell cycle which may affect the loci included in the epigenetic clocks^48,49^. Whether such “epigenetic drift” upon IH exposure occurs randomly across the genome or it affects only a specific set of loci, remains to be elucidated. Unlike high-turnover tissues, epigenetic aging in post-mitotic tissues, such left cardiac ventricle, is more likely driven by metabolic stress, DNA damage, or inflammation rather than replication^50–52^. To fully understand this phenomenon, future studies will need compare differences in the trajectory of epigenetic aging in different tissues upon IH exposures.

There is an increasing number of reports on changes in DNA methylation associated with OSA or its modelling by IH in animal models, as recently summarized in^53^. In addition to the differences in epigenetic age acceleration, we found DNA methylation loci that are associated with several pathophysiological pathways for OSA. To account for the impact of the IH exposure duration, we created regression models with the type and duraiton of the exposure as variables. While IH-induced differential methylation in PBMCs was associated with a variety of molecular and cellular mechanisms related to the activation of intracellular signaling pathways, DMPs in left cardiac ventricle were mainly associated with cardiac dysfunction (Figure 4). The overlap between cardiac dysfunction pathways and senescence networks suggests that epigenetic dysregulation links cardiovascular disease and cellular senescence (Figure 4B). We have previously shown that the elimination of senescence cells by a pharmacological intervention enhances the reversibility of end-organ morbidities with treatment of OSA^12^, yet the identification of specific cellular pathways altered by IH exposure may bring novel opportunities for the development of more specifically targeted interventions.

### IH induces cellular senescence mechanisms. Ablation of p16^high^ cells ameliorates IH-induced cardiovascular dysfunction

Cellular senescence is starting to be recognized as a major pathophysiological mechanism in OSA and its co-morbidities^54–56^. Rather than being merely a passive byproduct of chronological aging, OSA accelerates the molecular aging process, leading to premature senescence in tissues and increased prevalence of age-related disorders such as CVD and diabetes^54^. Many OSA-induced cellular mechanisms drive cells to a senescent state, such as oxidative stress, chronic inflammation, the adoption of a senescence-associated secretory phenotype (SASP), circadian clock disruption, stem cell exhaustion, and mitochondrial dysfunction^11,54,55,57,58^. In this work, we showed that IH induces DNA methylation changes in key genes of these biochemical pathways and molecular network at tissue specific (i.e. left cardiac ventricle) and systemic (i.e., PBMC) levels (Figure 4), suggesting a major role of epigenetic dysregulation in the induction of senescence by OSA in the cardiovascular system.

We determined that epigenetic dysregulation of the *Cdkn2a* gene (i.e., encoding the p16 protein) plays a central role among the overlapping networks associated with cardiovascular dysfunction and cellular senescence. p16 is an inhibitor of cyclin-dependent kinases and the cell cycle that slows cell division and constitutes a prototypical marker of senescent cells in both mice and humans^38^. It has been shown that selective elimination of p16^high^ senescent cells lengthens the healthy lifespan and ameliorates various age-related disorders^59–63^. Here, we utilized transgenic models to localize and ablate p16^high^cells^23^. Ablation of p16^high^ cells in IH-exposed mice resulted in a reduction of blood pressure and restored the coronary flow reserve to levels comparable to RA controls (Figure 5). Noteworthy, at the molecular level, cellular senescence involves the activation of several key signaling pathways and networks that converge on p53/p21 and p16/Rb axes^64–66^. We reason that IH might also induce other molecular networks leading to senescent cellular phenotypes (e.g., p53/p21 axis). Hence, further investigation is warranted, especially in terms of epigenetic dysregulation and possible synergies in the pathophysiology of OSA and cardiovascular comorbidities.

This study has some limitations that will need to be addressed in future studies. First, we focused our work on male mice. Female mice were reported to be relatively protected from IH-induced hypertension and tachycardia, suggesting sex differences in blood pressure responses to IH^67^. Using only male mice enabled the combined analysis of phenotype and epigenotype, minimizing the impact of sex as a confounding factor. Future studies will be dedicated to the impact of the IH-induced epigenetic dysregulation in the anticipated sexual dimorphism. Second, we targeted selected components of the vascular system (i.e., left cardiac ventricle. Aorta and coronary vascular bed). Determining the impact of IH-induced epigenetic dysregulation and cellular senescence in other vascular networks will require further investigation.

In conclusion, we showed the major role of IH in inducing aging and promoting cellular senescence in the cardiovascular system in leading to CVD morbidity in OSA. By using transgenic mice, we delineated the impact of IH on p16 expression, systemic and/or tissue-specific aging and vascular senescence, along with epigenomic changes. Furthermore, we provide initial evidence demonstrating that systemic ablation of p16^high^ cells will revert OSA-induced vascular dysfunction, thereby pointing to potential development of therapeutic targets and translation to clinical settings. Taken together, our study creates a novel and innovative framework in OSA, by mechanistically demonstrating the causal impact of cellular senescence on OSA-induced cardiovascular dysfunction.

## Supplementary Files

This is a list of supplementary files associated with this preprint. Click to download.

- CorteseSupplementaryTableS1.xlsx
- CorteseSupplementaryTableS2.xlsx
- CorteseSupplementaryTableS3.xlsx
- CorteseSupplementaryTableS4.xlsx
- CorteseSupplementaryTableS5.xlsx

## Data Availability

To ensure independent interpretation of clinical study results and enable authors to fulfill their role and obligations under the International Committee of Medical Journal Editors (ICMJE) criteria, the authors grant all external authors access to data pertinent to the development of the publication upon request. Scientific and medical researchers can request access to molecular and physiological data utilized in this publication.
